# Dielectric Characterization of Solutions of Galactomannan Extracted from *Adenanthera pavonina* L.: Effects of Purification and Ethanol Concentration

**DOI:** 10.3390/polym16111476

**Published:** 2024-05-23

**Authors:** Susana Devesa, Manuel P. F. Graça, Walajhone O. Pereira, Guilherme L. Santos, João F. da Silva Neto, Filipe M. B. Amaral, Imen Hammami, Fernando Mendes, Ana A. M. Macêdo

**Affiliations:** 1 CEMMPRE, ARISE, Department of Mechanical Engineering, University of Coimbra, Rua Luís Reis Santos, 3030-788 Coimbra, Portugal; 2 I3N and Physics Department, University of Aveiro, 3810-193 Aveiro, Portugal; filipe.amaral@estesc.ipc.pt (F.M.B.A.); imenhammami@ua.pt (I.H.); 3 Instituto Federal do Maranhão (IFMA)—Campus Imperatriz, Imperatriz 65906-335, MA, Brazil; walajhone.oliveira@ufma.br (W.O.P.); guilhermelima1000@hotmail.com (G.L.S.); netoferreira@acad.ifma.edu.br (J.F.d.S.N.); anaangellica@yahoo.com.br (A.A.M.M.); 4 Polytechnic Institute of Coimbra, Coimbra Health School (ESTeSC), 3046-854 Coimbra, Portugal; 5 Polytechnic University of Coimbra, ESTESC, UCPCBL, 3046-854 Coimbra, Portugal; fjmendes@estesc.ipc.pt; 6 H&TRC—Health and Technology Research Center, Coimbra Health School, Polytechnic University of Coimbra, 3046-854 Coimbra, Portugal; 7 Biophysics Institute of Faculty of Medicine, Coimbra Institute for Clinical and Biomedical Research (iCBR) Area of Environment Genetics and Oncobiology (CIMAGO), University of Coimbra, 3000-548 Coimbra, Portugal; 8 Center for Innovative Biomedicine and Biotechnology, University of Coimbra, 3000-548 Coimbra, Portugal; 9 Clinical Academic Center of Coimbra, Coimbra, Portugal, Polytechnic University of Coimbra, ESTESC, UCPNS, SM Bispo, 3045-093 Coimbra, Portugal; 10 European Association for Professions in Biomedical Sciences, 1000 Brussels, Belgium

**Keywords:** *Adenanthera pavonina* L., Galactomannan, dielectric properties, impedance spectroscopy

## Abstract

Galactomannans are polysaccharides obtained from legume seed extraction. They present a chemical structure consisting of D-mannose chains linked by glycosidic bonds and galactose branches. The main focus lies in their use as thickeners in the food industry, aimed at improving the dielectric properties of food during heating processes within the radiofrequency and microwave ranges. In this work, the prepared galactomannan samples were electrically analyzed through impedance spectroscopy, which is a powerful physical technique. From the experimental measurements, the dielectric permittivity and loss tangent of the galactomannan solutions were analyzed and the electrical modulus formalism was used to study the dielectric relaxations. Crude galactomannans exhibited higher values of permittivity, conductivity, and losses compared to purified galactomannans. Increasing ethanol concentration in galactomannan purification causes an increase in the permittivity and conductivity of galactomannan solutions. In a 1% solution, at 1 kHz, the permittivity increased from 378.56 to 538.09, while in the 2% solution, this increase was from 656.22 to 1103.24. Regarding the conductivity, at the same frequency, the increase was from 1.6 × 10^−3^ to 3.3 × 10^−3^ Ω^−1^m^−1^ and from 2.9 × 10^−3^ to 5.5 × 10^−3^ Ω^−1^m^−1^, respectively. The rise of the ethanol concentration in galactomannan purification led to a decrease in the relaxation time, from 448.56 to 159.15 μs and from 224.81 to 89.50 μs in the solution with 1 and 2%, respectively. The results suggest that galactomannan from *Adenanthera pavonina* L. has potential for use in the food industry.

## 1. Introduction

Some emerging technologies in the food industry, e.g., radiofrequency and microwave heating, hold significant potential for replacing conventional heating methods, which involve heat transfer by convection or conduction. These new techniques allow for the more homogeneous heating of food. Understanding the dielectric properties of the raw materials used in food production is essential for comprehending variations in heating rates [1,2,3].

The dielectric properties of materials play a crucial role in how they interact with electromagnetic energy. When subjected to intense radiofrequency or microwave electric fields, these properties determine the extent of dielectric heating. Understanding these interactions provides valuable insights for microwave or radiofrequency processing, especially in the realm of food products. Additionally, this knowledge is essential for devising effective and consistent pasteurization methods and for selecting the most suitable frequency ranges for radiofrequency and microwave heating [4].

Dielectric properties are often separated into two categories: dielectric constant and dielectric losses. The dielectric constant is related to the ability of a material to store energy and the dielectric losses to the ability of a material to dissipate energy into heat. These properties are of prime importance regarding the radiofrequency and microwave processing of non-metallic materials, such as polymers, where low dielectric losses result in minimal heating [4,5].

**Figure 1:**
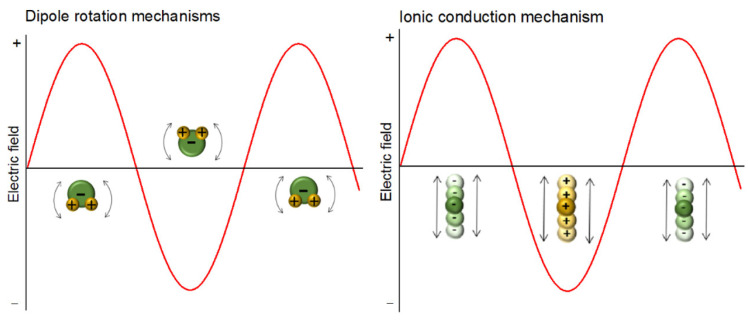
Radiofrequency and microwave heating mechanisms.

When subjected to electromagnetic radiation in the radiofrequency and microwave ranges, dielectric materials undergo heating through the conversion of electric energy into heat. In the radiofrequency and microwave heating process, two major mechanisms, shown in Figure 1, should be considered: dipolar polarization, for dipoles, and ionic conduction, for ions. In the dipolar polarization, the electric field exercises a torque on the electric dipole. Molecules with dipole moments, such as water molecules, attempt to align these dipole moments with the electric component of the electromagnetic field, so they are continuously rotating. This movement produces thermal energy through molecular friction and dielectric loss. In the ionic conduction mechanism, ions, cations or anions, oscillate back and forth within the electric field and their collisions with each other generate heat [6,7].

It is important to highlight that the dipolar polarization mechanism primarily contributes to heat generation in the liquid phase, whereas the ionic conductivity mechanism predominates in solid materials [6].

Also, it is crucial to differentiate between radiofrequency and microwave technologies. Despite their proximity on the electromagnetic spectrum in terms of wavelength, in radiofrequencies, an electric field is generated between electrodes, whereas in microwaves, it involves the propagation and reflection of waves governed by the laws of optics [7].

This heating method proves more efficient than conventional approaches, requiring less processing time and, therefore, enhancing the final product quality while reducing treatment costs [2,8].

Factors such as moisture content, temperature, density, and applied frequency strongly influence the dielectric properties of materials and, consequently, the mechanisms of heating through electromagnetic radiation in the radiofrequency and microwave ranges. This heating system has been used in various food industry processes, such as pasteurization, sterilization, and enzymatic inactivation [2]. Water and salts are also factors that influence dielectric heating. Notably, molecules possessing permanent dipoles in their composition, such as galactomannans, can contribute to an enhanced increase in heating rates [9,10]. Thus, it is important to know the dielectric properties of polymers that can be incorporated into food, seeking to understand the interaction between electromagnetic field energy and food, which can make electromagnetic radiation heating methods more effective.

In addition to its role in the food sector, galactomannan, due to its distinctive physical-chemical characteristics, finds applications in various other industries including pharmaceuticals, biomedicine, and cosmetics, where it is used as a thickener, stabilizer, encapsulator, etc. In the electronics industry, dielectric polymers have extensive applications, such as being used as dielectrics in capacitors [11]. According to the European Semiconductor Industry Association (ESIA), dielectrics hold significant importance in the electronic industry market, reaching USD 42.597 billion in Europe in 2018 and a global total of USD 468.778 billion [12]. Therefore, understanding the electrical properties of polymers is crucial, including galactomannan from *Adenanthera pavonina* Linnaeus.

Polymers can be either natural or synthetic materials with high molecular weight, characterized by a chemical structure composed of carbon, hydrogen, and non-metallic elements [13]. While natural polymers can be found in nature, synthetic polymers are obtained via polymerization reactions. Natural polymers include lipids, proteins, and polysaccharides, among other substances, and find applications in various fields such as the food industry, civil construction, electronics, and biomedicine [14,15]. Due to the growing importance of renewable and environment-friendly materials, cellulose, which is also a natural polysaccharide, has become a focal point of extensive research within worldwide industrial companies [16].

**Figure 2:**
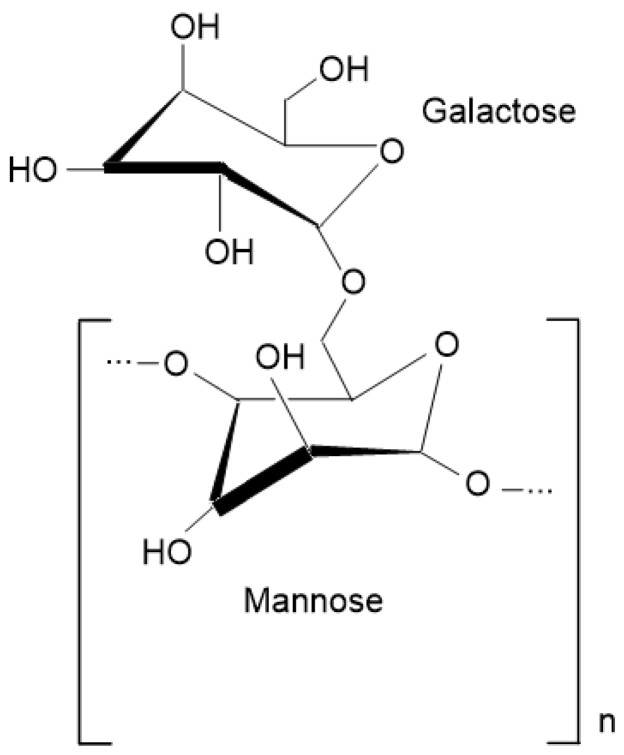
Galactomannan chemical structure (ACD/ChemSketch 2023, version 2.02.0, Advanced Chemistry Development, Inc., Toronto, Canada).

Polysaccharides can consist of one or more types of monosaccharides. Galactomannans are an example of neutral polysaccharides, formed by the combination of mannose and galactose. Their general structure (Figure 2) comprises D-mannopyranose units linked by β-(1→4) glycosidic bonds and D-galactopyranosyl units joined by α-(1→6) glycosidic bonds [17]. Galactomannans are found in Fabaceae (Legumosae) seed endosperms and in smaller quantities in the Palmae, Annonaceae, Convolvulaceae, Ebenaceae, and Loganiaceae families, as well as in certain fungi and lichens [15,18,19]. The ratio of mannose/galactose in galactomannans varies depending on the species and environmental conditions, with values ranging between 1.0 and 5.0. These variations are important as they affect several physicochemical properties of galactomannans, including solubility, enzymatic degradation, gelation, and molecular interactions. Cerqueira et al. [20] reported the M/G ratio for galactomannan extracted from *A. Pavonina* as 1.8.

In this work, galactomannan was extracted from *Adenanthera pavonina* L. and purified by the ethanol precipitation method.

There are several ways to achieve the purification of crude galactomannan samples; however, the precipitation with ethanol has been largely used, due to its advantages [21]. It is simpler and easier to perform when compared to other existing extraction and purification methodologies, it is able to generate food-grade substances, it avoids the use of organic solvents rather than ethanol, and it is environmentally friendlier. Another factor that should be taken into consideration is the yield of the purification process since it is one of the most economically important aspects of polysaccharide extraction and purification [20]. Cerqueira et al. [20], using ethanol purification on galactomannan extracted from three different species of Leguminosae, *Adenanthera pavonina*, *Caesalpinia pulcherrima*, *and Gleditsia triacanthos*, achieved yields of purification between 42 and 66%.

Galactomannans have potential for various industrial applications, such as being used as conjugated polymers exhibiting semiconductor behavior linked to molecular π orbits translated along the polymer chain. These polymers have recently found use in biosensing, imaging, and other biomedical applications due to their strong light-harvesting capabilities, high fluorescence quantum yield, and excellent photostability [22,23]. Several foods have undergone analysis of their dielectric properties across different temperature ranges, including grape juices, pineapple, apple, pear, and orange, as well as honey, milk, and soy beverages [3,8,24]. Due to its ability to form solutions with high viscosities in small proportions, galactomannan can still be used as a thickener when incorporated into food [14,15], and it can also improve its dielectric properties.

In this study, the dielectric properties of galactomannan solutions were investigated to gain a better understanding of their behavior when exposed to electric fields in the radiofrequency and microwave ranges, for potential applications in the food industry.

## 2. Materials and Methods

### 2.1. Preparation Methods

#### 2.1.1. Extraction of Galactomannan from *Adenanthera pavonina* L.

**Figure 3:**
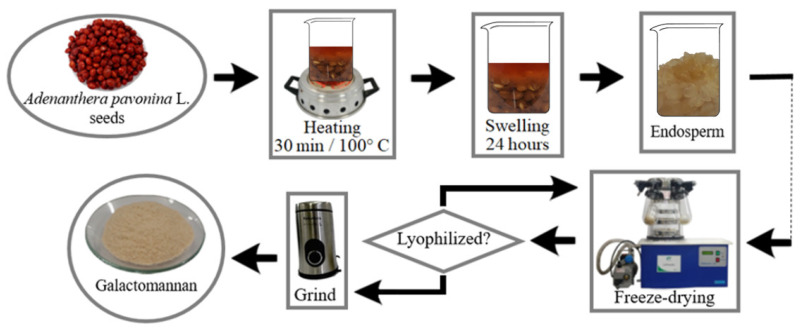
Extraction flow chart of galactomannan from *Adenanthera pavonina* L.

The seeds were heated with distilled water at 100 °C for 30 min, with an initial volume ratio of 1:3, and then swollen for 24 h at room temperature. Subsequently, the seeds were washed with distilled water and the endosperm was separated from the embryo and integument. Then, the endosperms were lyophilized in the Liotop model L101 (Liobras, São Paulo, Brasil) lyophilizer. The material was then sprayed onto the Taurus Aromatic (Bainbridge, WA, USA) model crusher and sieved on a 250 μm mesh (Figure 3).

#### 2.1.2. Purification of Galactomannan from *Adenanthera pavonina* L. in Different Concentrations

To purify crude galactomannan, the endosperm is triturated with distilled water in a 1:1 volume ratio and then sieved. The obtained solution was divided into five parts for the precipitation of galactomannan in ethyl alcohol. For this step, ethanol solutions were prepared at varying concentrations of 60%, 70%, 80%, 90%, and 100% (V/V) using 95% P.A. ACS ethyl alcohol from Vetec (Cotia-SP, Brazil). Consequently, the galactomannan was purified in the ethanol solutions at the specified concentrations, yielding the GP60, GP70, GP80, GP90, and GP100 samples, respectively. Subsequently, all samples were dried at 50 °C for 24 h using a QUIMIS model Q314M-222 oven (Diadema, SP, Brazil).

#### 2.1.3. Preparation of Galactomannan Solutions from *Adenanthera pavonina* L.

*Adenanthera pavonina* L. crude galactomannan solutions were prepared at concentrations of 1 and 2% (*wt*/*wt*), referred to as samples GB 1% and GB 2%, respectively, and purified galactomannan solutions were prepared at the same concentrations of 1 and 2% (*wt*/*wt*). Thus, the samples GP60 1% and GP60 2% were obtained from the 60% ethanolic solution purification process. The same process was adopted for the remaining purification concentrations, 70, 80, 90, and 100%. After, all the solutions were left for 24 h at room temperature on a magnetic stirrer and then centrifuged for 30 min using a benchtop centrifuge DAIKI model 80-2B (Osaka, Japan).

### 2.2. Characterization Methods

#### 2.2.1. Infrared Spectroscopy

The structure of the samples was analyzed using infrared spectroscopy. Measurements were performed on a PerkinElmer Spectrum Two spectrometer system (PerkinElmer, Hopkinton, MA, USA), in the wavelength range of 8300–350 cm^−1^. For this, pellets of the GP60, GP70, GP80, GP90, and GP100 samples with 95% of potassium bromide (KBr) were prepared using a uniaxial pressure system combined with a primary vacuum pump. All the measurements were performed at room temperature.

#### 2.2.2. Electrical Measurements

**Figure 4:**
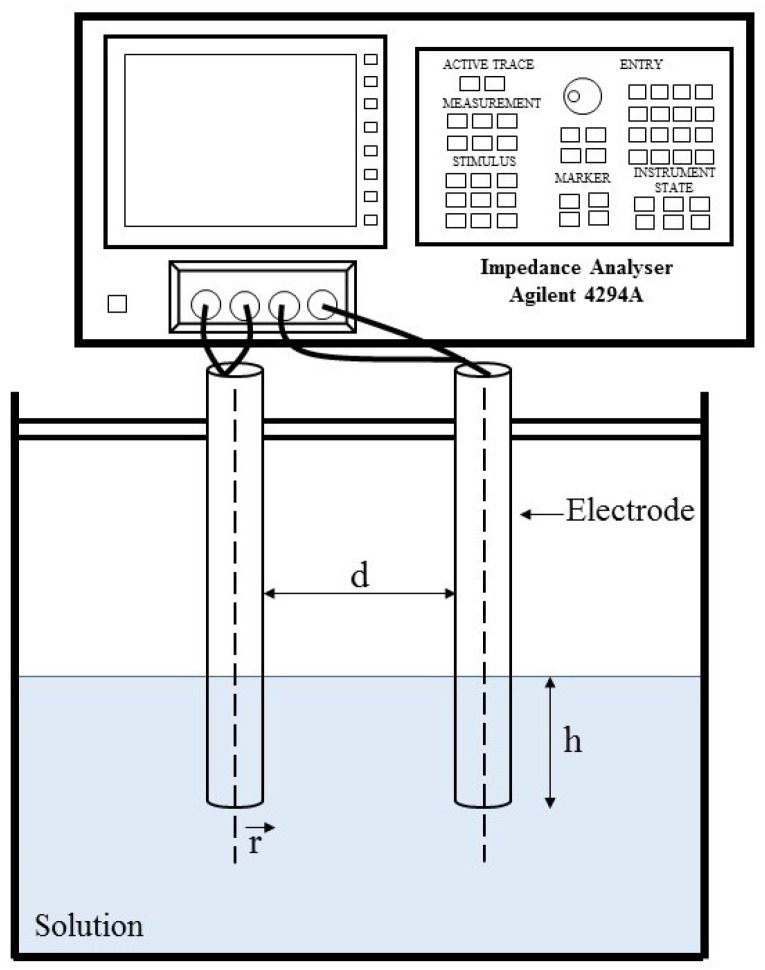
Schematic diagram of the solution dielectric measurement configuration (adapted from Tagmouti et al. [25]).

AC electrical measurements were performed using an Agilent 4294A impedance analyzer (Santa Clara, CA, USA), in the frequency range from 100 Hz up to 1 MHz, at room temperature. The method proposed by Tagmouti et al. [25], illustrated in Figure 4, was adopted for these characterizations. Initially, each solution was placed in a container containing two electrodes of equal radius (r), separated by a fixed and known distance (d), and kept submerged at a certain height (h). The impedance analyzer was used in the Cp-Rp configuration, i.e., performing equivalent measurements of a capacitor Cp in parallel with a resistor Rp.

The dielectric permittivity is given as a complex variable (ε*), which can be divided into the real part (ε′) and imaginary part (ε″). The real and imaginary components of the complex permittivity can be deduced from the measured data of C_p_ and R_p_ by using Equations (1) and (2) [25,26]:

(1) $$\varepsilon^{\prime }=\frac{C_{p}}{\pi \varepsilon_{0}h}ln\left[\frac{d}{r}+\sqrt{{\left(\frac{d}{r}\right)}^{2}-1}\right]$$

(2) $$\varepsilon^{\prime \prime }=\frac{1}{\pi \varepsilon_{0}R_{p}\omega h}ln\left[\frac{d}{r}+\sqrt{{\left(\frac{d}{r}\right)}^{2}-1}\right]$$

where ε_0_ is the dielectric constant of vacuum air, with a value of 8.854 × 10^−12^ F/m, and ω is the angular frequency.

The loss tangent (tg δ), presented in Equation (3), relates the material’s ability to dissipate energy (ε″) in the form of heat with the storing energy (ε′) when subjected to an external electric field.

(3) $$tan\;\delta =\frac{\varepsilon^{\prime \prime }}{\varepsilon^{\prime }}$$

The AC conductivity is obtained from the empirical relationship presented in Equation (4) [27,28]:

(4) $$\sigma_{AC}=\omega \varepsilon_{0}\varepsilon^{\prime \prime }$$

Equation (5) presents the mathematical formalism between the permittivity and the electric modulus (M*). This formalism is commonly used in dielectric analysis as it minimizes the effect of the electrode–sample interface capacitance at low frequencies and emphasizes small features at high frequencies. Thus, the sample bulk dielectric behavior can be easily observed [29,30,31].

(5) $$M^{*}=\frac{1}{\varepsilon^{*}}=\frac{1}{\varepsilon^{\prime }-j\varepsilon^{\prime \prime }}=\frac{\varepsilon^{\prime }}{{\varepsilon^{\prime }}^{2}+{\varepsilon^{\prime \prime }}^{2}}+j\frac{\varepsilon^{\prime \prime }}{{\varepsilon^{\prime }}^{2}+{\varepsilon^{\prime \prime }}^{2}}=M^{\prime }+jM^{\prime \prime }$$

where M′ and M″ are the real and imaginary parts of the electric modulus, respectively.

**Figure 5:**
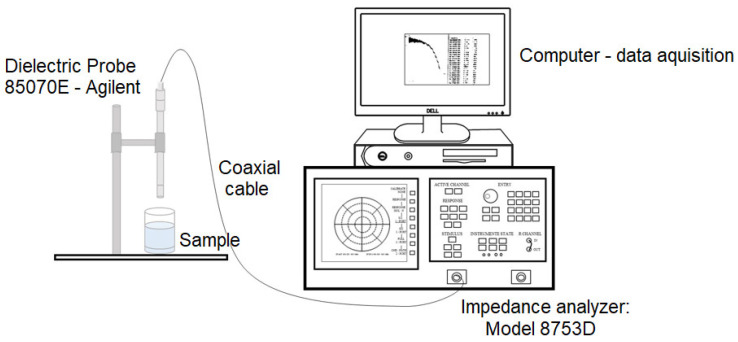
Scheme of the equipment for dielectric measurements in the microwave region (adapted from Everard et al. [32]).

The electrical measurements in the microwave region were performed using an Agilent 85070E (Agilent Technologies, Santa Clara, CA, USA) probe connected to a Hewlett Packard 8753D Network analyzer (Agilent Technologies), in the frequency range from 500 MHz to 6 GHz, at room temperature. For these measurements, the coaxial cable technique was employed where a sensor was connected to the impedance analyzer, and its mounting configuration is illustrated in Figure 5. The electrical characteristics were determined from the reflection coefficient of the sample in contact with the sensor. Measurements can be expressed in terms of relative permittivity, complex relative permittivity, and the dielectric loss factor.

The equipment needs to be calibrated using a dedicated calibration kit. During the calibration process, the sensor measures the relative permittivity of the air, and then the relative permittivity of the deionized water is determined at a known temperature. With the calibrated equipment, it was possible to perform tests with C_2_H_5_OH and distilled water to verify measurement accuracy. The results were compared with values from the literature.

## 3. Results and Discussion

**Figure 6:**
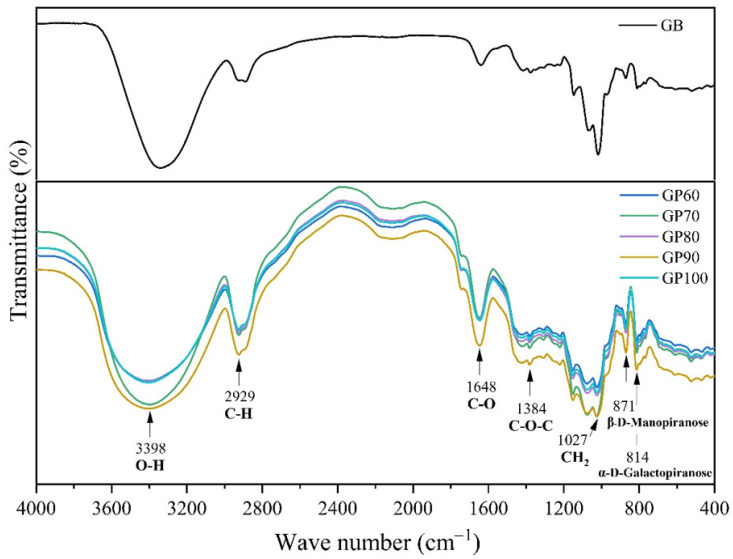
FTIR spectra of the GB, GP60, GP70, GP80, GP90, and GP100 samples.

Figure 6 shows the Fourier-transform infrared (FTIR) spectra of the GB, GP60, GP70, GP80, GP90, and GP100 samples. The absorption band observed at 3398 cm^−1^ corresponds to the vibrational stretch of hydroxyl groups, along with water involved in the formation of hydrogen bridges [33]. The absorption band at 2929 cm^−1^ is related to the stretching of the group -CH. The vibration at 1648 cm^−1^ is attributed to the stretching vibration of the galactose and mannose rings. The vibration observed at 1384 cm^−1^ is related to the coupled absorption modes of the polymeric skeleton [34]. The band at 1027 cm^−1^ is attributed to the vibrational displacement of the -CH_2_ group [35], while the two absorption bands at 871 cm^−1^ and 812 cm^−1^ are associated with the presence of α-D-galactopyranose and β-D-manopyranose units, respectively [36]. The slight changes observed in the spectra result from the formation of new hydrogen bonds caused by the purification of galactomannan. As expected, all the samples exhibit similar spectra, indicating that the purification process did not significantly alter the structure of galactomannan.

**Figure 7:**
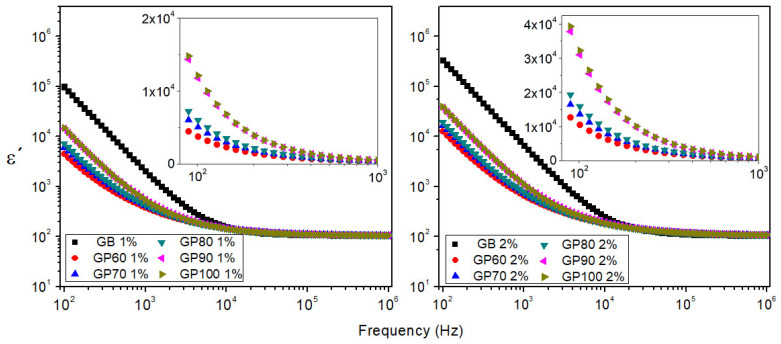
Real part of dielectric permittivity as a function of frequency of 1% and 2% solutions for GB, GP60, GP70, GP80, GP90, and GP100 samples at room temperature (inset shows the low-frequency region).

Figure 7 presents the real part of the dielectric permittivity (*ε*′) as a function of the frequency for the GB, GP60, GP70, GP80, GP90, and GP100 samples, at room temperature. It can be seen for all samples that the real part of the dielectric permittivity decreases as frequency increases. This phenomenon is attributed to the dipole relaxation effect because, at low frequencies, the dipoles easily align with the applied electric field, while this does not occur in the high-frequency region [37]. The values of *ε*′ are relatively high. One possible justification can be related to permanent carbonyl dipoles from galactose and mannose molecules in galactomannan that, in a cooperative effect, can induce other dipoles to align with the electric field by conformational variations [38]. Figure 7 shows, in the inset, the low-frequency region, where it can be seen that for purified galactomannan samples, the real part of the dielectric permittivity increases with increasing alcohol concentration. Similar behavior is observed in the AC conductivity, loss tangent, and imaginary part of the modulus function. This behavior probably occurs because alcohol disrupts galactomannan’s intramolecular hydrophobic interactions, causing its dehydration with increasing ethanol concentration [39].

**Figure 8:**
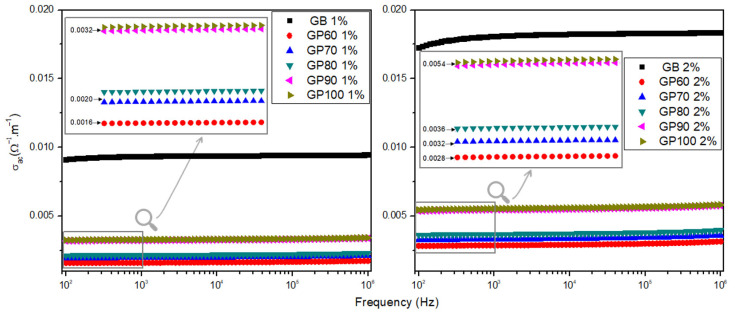
AC conductivity as a function of the frequency of 1% and 2% solutions for GB, GP60, GP70, GP80, GP90, and GP100 samples at room temperature.

Figure 8 shows the AC conductivity as a function of the frequency of GB, GP60, GP70, GP80, GP90, and GP100 samples measured at room temperature. It is noted that the conductivity tends to increase as the solution concentration increases, which may be related to the increase in the number of charge-bearing particles present in galactomannan. This can be because the alcohol causes the dehydration of galactomannan molecules, and therefore as the ethanol concentration increases, the polymer chain’s rigidity rises due to increased stiffness in hydrogen bonds, leading to a limitation in polymer chain mobility [40].

**Figure 9:**
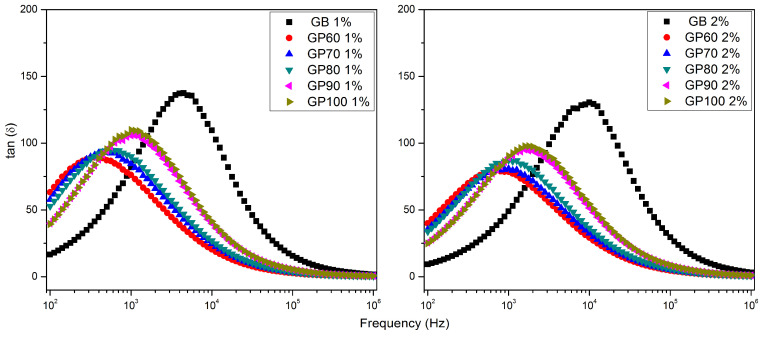
Loss tangent as a function of frequency of 1% and 2% solutions for GB, GP60, GP70, GP80, GP90, and GP100 samples at room temperature.

The loss tangent as a function of the frequency of samples GB, GP60, GP70, GP80, GP90, and GP100 the of 1 and 2% solutions at room temperature is shown in Figure 9. It is visible that the dielectric loss is greater than 1 for all samples, which implies, according to Equation (3), that the dissipated energies are greater than the energies stored in this frequency range. A maximum in *tan δ* is observed in all samples. For all samples, the maximum shifts to higher frequencies with the increase in the load from 1 to 2%. In both series, the sample GB exhibits this peak at the highest frequency. It is also visible that an increase in ethanol concentration results in a shift of this peak towards higher frequencies. These maxima should be related to a dielectric relaxation phenomenon.

**Figure 10:**
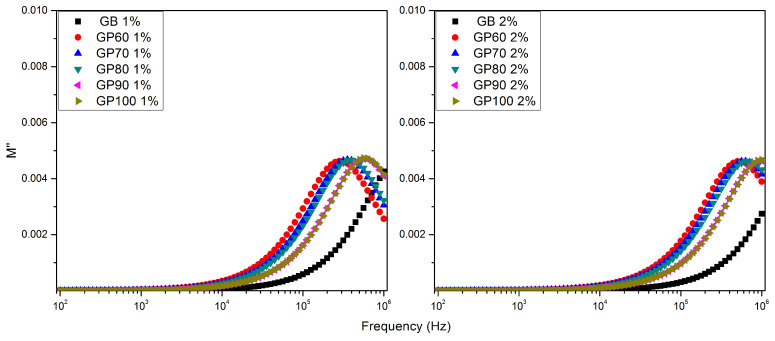
Imaginary part of the electric modulus as a function of the frequency of 1% and 2% solutions for GB, GP60, GP70, GP80, GP90, and GP100 samples at room temperature.

Figure 10 shows the imaginary part of the modulus formalism (*M*″) as a function of frequency for the samples GB, GP60, GP70, GP80, GP90, and GP100 at room temperature. The patterns indicate the presence of a new dielectric relaxation phenomenon at high frequencies, which shifts towards higher frequencies with an increase in ethanol concentration. This is the same trend observed in the relaxation phenomena revealed using the loss tangent formalism. This leads to the hypothesis that, in the high-frequency range, the ions may be spatially confined within potential wells, performing localized motions [41] and promoting localized polarizations.

Table 1 and Table 2 summarize the electrical parameters, obtained at 1 kHz and room temperature, and the frequency corresponding to the *tan δ* and *M*″ peaks, with the respective relaxation times, *τ* = (2π*f*)^−1^, for the 1% and 2% solutions of the samples GB, GP60, GP70, GP80, GP90, and GP100.

The observed trend indicates that as the percentage of galactomannan in the solution increases, there is a corresponding rise in the dielectric constant. Simultaneously, the loss tangent experiences, in general, a decrease. This shift is accompanied by a reduction in relaxation times, implying that peaks in the representation of the loss tangent and imaginary part of the modulus occur at higher frequencies with increasing concentration of the solution.

**Figure 11:**
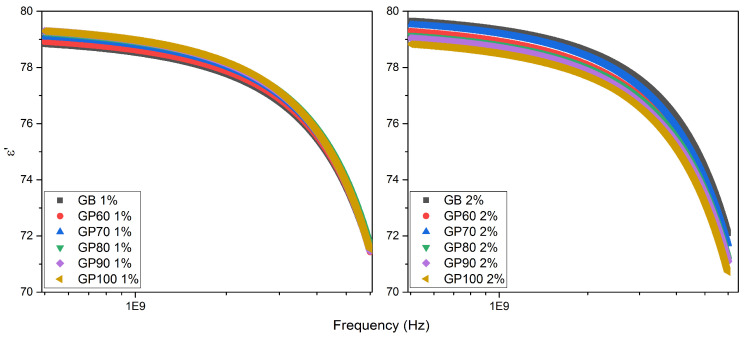
Relative permittivity as a function of frequency of 1% and 2% solutions for GB, GP60, GP70, GP80, GP90, and GP100 samples at room temperature.

Figure 11 shows the experimental results of the relative permittivity of the samples GB, GP60, GP70, GP80, GP90, and GP100, in the range of 500 MHz to 6 GHz. It is observed that the permittivity decreases as the frequency increases; this may be related to the fact that with the increase in the frequency of oscillation of the electric field, polar molecules do not follow the abrupt changes of the external field polarity. The values found for relative permittivity are close to 80, a fact that may be associated with the solvent used [42].

**Figure 12:**
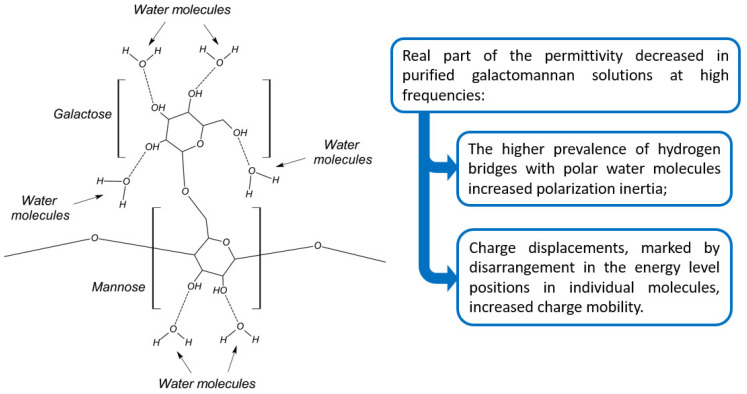
Schematic representation of the correlation between the chemical structure and the real part of the permittivity in purified galactomannan solutions [40,43,44].

Figure 12 shows, schematically, the correlation between the chemical structure of the polymeric solutions and the real part of the permittivity.

## 4. Conclusions

Galactomannan was successfully extracted from *Adenanthera pavonina* L. and subjected to a purification process using various ethanol concentrations. Subsequently, galactomannan solutions were prepared at concentrations of 1 and 2% (*wt*/*wt*).

Structural analysis using FTIR spectroscopy showed that after purification, the galactomannan preserves its characteristic monosaccharides, such as mannose and galactose.

From the impedance spectroscopy measurements, it was concluded that crude galactomannan presented the highest values for permittivity, conductivity, and losses. This behavior could be attributed to material impurities. On the other hand, purified galactomannans exhibited enhanced dielectric properties as the ethanol concentration used in the purification process increased. The solutions present a stable conductivity across a broad frequency range, characteristics that imply possible application in the food industry, as well as in biosensing, imaging, and other biomedical applications.

## Figures and Tables

**Table 1:** Electrical parameters for the 1% solutions of the samples GB, GP60, GP70, GP80, GP90, and GP100.

	*ε*′	*tan δ*	σ_ac_ (Ω^−1^m^−1^)	*f* (*tan δ* _max_) (Hz)	*τ* (*tan δ*) (μs)	*f* (*M*″ _max_) (Hz)	*τ* (*M*″) (μs)
	*f* = 1 kHz						
GB 1%	2051.34	81.90	0.0093	4467	35.63	-----	-----
GB60 1%	378.56	76.17	0.0016	355	448.56	281,838	0.56
GB70 1%	419.88	84.57	0.0020	447	356.30	354,813	0.45
GB80 1%	429.75	89.98	0.0022	562	283.02	398,107	0.40
GB90 1%	547.53	105.81	0.0032	1000	159.15	562,341	0.28
GB100 1%	538.09	109.82	0.0033	1000	159.15	562,341	0.28

**Table 2:** Electrical parameters for the 2% solutions of the samples GB, GP60, GP70, GP80, GP90, and GP100.

	*ε*′	*tan δ*	σ_ac_ (Ω^−1^m^−1^)	*f* (*tan δ* _max_) (Hz)	*τ* (*tan δ*) (μs)	*f* (*M*″ _max_) (Hz)	*τ* (*M*″) (μs)
	*f* = 1 kHz						
GB 2%	6624.99	49.09	0.0181	10,000	15.92	-----	-----
GB60 2%	656.22	78.58	0.0029	708	224.81	501,187	0.32
GB70 2%	734.77	80.90	0.0033	1000	159.15	630,957	0.25
GB80 2%	757.95	87.12	0.0037	1259	126.42	707,946	0.22
GB90 2%	1096.66	89.24	0.0054	1585	100.42	-----	-----
GB100 2%	1103.24	90.28	0.0055	1778	89.50	-----	-----

## Data Availability

The raw data supporting the conclusions of this article will be made available by the authors on request.
